# Brain region specific regulation of anandamide (down) and sphingosine-1-phosphate (up) in association with anxiety (AEA) and resilience (S1P) in a mouse model of chronic unpredictable mild stress

**DOI:** 10.1007/s00424-024-03012-0

**Published:** 2024-08-23

**Authors:** Caroline Fischer, Dominique Thomas, Robert Gurke, Irmgard Tegeder

**Affiliations:** 1 Goethe-University Frankfurt, Faculty of Medicine, Institute of Clinical Pharmacology, Theodor-Stern-Kai 7, 60590 Frankfurt, Germany; 2 Fraunhofer Institute for Translational Medicine and Pharmacology (ITMP), Theodor-Stern-Kai 7, 60590 Frankfurt, Germany

**Keywords:** Sphingolipids, Endocannabinoids, Chronic unpredictable mild stress, Ceramides, Hippocampus, Prefrontal cortex, Thalamus, Midbrain, Anxiety, Depression, Resilience

## Abstract

Chronic unpredictable and unavoidable stress is associated with mental health problems such as depression and anxiety, whereas cycles of stress and stress relief strengthen resilience. It has been suggested that increased breakdown of brain endocannabinoids (eCB) promotes a feeling of adversity. To assess the impact of stress on bioactive lipid homeostasis, we analyzed eCB, sphingolipids, and ceramides in seven brain regions and plasma in a mouse model of chronic unpredictable mild stress. Chronic unpredictable mild stress (CUMS) was associated with low levels of anandamide in hippocampus and prefrontal cortex in association with indicators of anxiety (elevated plus maze). Oppositely, CUMS caused elevated levels of sphingosine-1-phosphate (S1P d18:1) and sphinganine-1-phosphate (S1P d18:0) in the midbrain and thalamus, which was associated with readouts of increased stress resilience, i.e., marble burying and struggling in the tail suspension tests. In the periphery, elevated plasma levels of ceramides revealed similarities with human major depression and suggested unfavorable effects of stress on metabolism, but plasma lipids were not associated with body weight, sucrose consumption, or behavioral features of depression or anxiety. The observed brain site-specific lipid changes suggest that the forebrain succumbs to adverse stress effects while the midbrain takes up defensive adjustments.

**Supplementary Information:**

The online version contains supplementary material available at 10.1007/s00424-024-03012-0.

## Introduction

Stress exposure is normal in daily life, but it is also a predisposing risk factor for metabolic, cardiovascular, and mental disease such as anxiety and depression [13, 31, 95]. Stressors do not influence all individuals equally. Some are vulnerable while others are more resilient [32, 33]. The adaptations of the brain to acute and short-lasting stressful conditions involve neuronal systems that also process rewarding stimuli [2, 63, 104]. Studies indicate that the ability to cope with acute or chronic stress relies in part on the neurotransmitter, dopamine, mostly considered as a signal of reward or stress relief [20, 32, 64]. However, dopamine is also involved in aversive motivation [47, 86]. Acute stress increases extracellular dopamine levels in the mesolimbic mesocortical dopamine system, particularly the nucleus accumbens and prefrontal cortex (PFC) [2, 93], but chronic or intense stressors, particularly prolonged, repetitive, unpredictable, and unavoidable stress rather inhibit dopamine release or dopamine receptor responsiveness [64, 82, 101, 107]. In rodents, chronic restraint stress, chronic social defeat stress, and chronic unpredictable mild stress (CUMS) are associated with depressive-like behaviors [22, 76] and are linked to suppressed midbrain dopaminergic neuronal activity [2, 86].

There is some evidence from previous studies that microbiome-derived lipids [97] and endogenous bioactive lipids including prostaglandins, sphingolipids, and endocannabinoids contribute to the mal-adaptations to chronic stress at the molecular level [54, 62, 72], and in turn, are regulated by chronic or acute stress [56, 89]. Ceramides are believed to aggravate or maintain aversiveness [54] and have been suggested as putative targets for antidepressants [8, 41, 78] whereas endocannabinoids (eCBs) protect against psychological sequelae of chronic stress [57], likely as positive indirect modulators of the dopamine system. DA neurons in the midbrain receive excitatory glutamatergic and inhibitory GABAergic input [74, 81, 100], and both are under inhibitory control via presynaptic cannabinoid receptors [10, 14, 35, 37]. Functions of sphingoid-base sphingolipids are unclear and mostly based on studies with FTY720 (fingolimod [21]), which acts as a sphingosine-1-phosphate (S1P) receptor agonist after phosphorylation but leads to receptor downregulation. One study revealed that S1P-dependent stress resilience required signaling through S1P receptor 3 (*S1PR3*) in the medial prefrontal cortex [19], but alternative effects such as inhibition of microglial inflammasome activation were also suggested [43].

The evidence of a mutual endocannabinoid-mediated stress-lipid signaling arises from preclinical studies with genetic ablation or pharmacologic antagonism of the cannabinoid type 1 receptor (*Cnr1*, CB1) [3, 4, 70], or from clinical experience with the CB1-antagonist, rimonabant [29]. Knockout of CB1 results in exaggerated neuroendocrine and behavioral responses to acute stress including anxiety, reward sensitivity, pain, morphologic changes in the amygdala, and hippocampal synaptic plasticity [58, 59, 69, 70, 94, 113]. Under acute stress, endocannabinoids limit the magnitude of the stress response and facilitate recovery after cessation of stress exposure [42, 69]. However, under chronic or severe stress, the endocannabinoid system appears to “collapse” in the sense that CB1 receptors are downregulated or dysfunctional or that endocannabinoids are missing [102]. Hence, the ability to modulate the synaptic release of neurotransmitters such as glutamate and gamma-aminobutyric acid is lost [52, 88, 103]. Consequently, inhibitors of endocannabinoid breakdown attenuated stress-evoked behavioral manifestations of depression or anxiety [5, 6, 42, 51]. In humans, posttraumatic stress disorder or major depressive disorder is associated with reductions of the circulating levels of endocannabinoids [48, 66, 106], and a significant proportion of individuals using the CB1 antagonist rimonabant for weight loss developed indices of anxiety and depression and suicide and finally, withdrawal of the drug [16, 99].

Based on these studies, we hypothesized that chronic stress leads to brain-region-dependent changes of endocannabinoids, which in turn would increase the suffering from chronic stress. The vicious cycle may be fixated by genetic or epigenetic modifications and further profound changes of connected or independent lipid signaling paths that as a whole determine the behavioral outcome and susceptibility to mental disease on one side and gain of resilience or even reward upon stress relief on the other side. To address this hypothesis, we analyzed lipids of emotion-relevant classes (endocannabinoids, ceramides, hexosylceramides, and sphingoid bases) in seven brain regions and plasma in association with behavior in a model of chronic unpredictable mild stress in mice.

## Methods

### Mice

Animal studies are reported and were conducted in compliance with the ARRIVE guidelines [84]. The experiments were approved by the local Ethics Committee for Animal Research (Darmstadt, Germany V 54—19 c20/15 FK1074) and adhered to the European guidelines and to those of GV-SOLAS for animal welfare in science. We used female mice which were raised in the local breeding facility. Matched pairs according to body weight and age were submitted to chronic unpredictable mild stress (CUMS; *n* = 11) and control groups (*n* = 10). Group assignment was blinded. The ages at the start ranged from 6 to 15 weeks (average 9.5 weeks) in both groups. The average weight at onset was 20.5 ± 0.7 g (CUMS) and 20.6 ± 1.2 g (control). Mice were housed in pairs (except one cage with *n* = 3) and kept in a controlled environment (12-h dark/light cycle, 23 °C, 55% humidity) with food and water ad libitum. Animal numbers were estimated by power analysis using GPower [34]. The probability of type-1 error was set to *α* = 0.05, and type-2 error was set at *β* = 0.2.

### Chronic unpredictable mild stress (CUMS)

To evoke mild stress, mice are exposed daily to different types of mild stressors, such as temporary isolation or crowded housing, tilted home cages, wet bedding, predator odor, cold exposure, restraint, itch, no food, no water, or disrupted dark–light cycle [83, 108]. The time of each stressor varies from 0.5 to 4 h per day or overnight, and the protocol can last for 3–12 weeks. The detailed protocol used in this study is provided as Supplementary timetable. To integrate behavioral tests of control mice, the stress protocol lasted for 6 weeks with a short break of 6 days without stressors. Behavioral tests of CUMS mice were integrated in the last stress exposure days. The stressors included cage switch, itch, cold exposure, TMT odor, headache (nitroglycerin i.p.), no bedding, wet bedding, lights on overnight, no food overnight, no water overnight, restraint, cage shaking, tilted cage, and ultrasound noise. The behavior tests using tail suspension and elevated plus maze per se are also considered stressors.

### Elevated plus maze (EPM)

The EPM takes advantage of the physiologic self-protective hiding of mice in the dark versus their curiosity. The test is considered to measure anxiety-like behavior. The standard EPM maze was configured with two orthogonally arranged closed arms (25 L × 5 W × 15 H cm) and two open arms (25 L × 5 W × 0.3 H cm) connected by a central open square (5 × 5 cm), and it was elevated 60 cm above the floor and illuminated from above. The floor and walls were made of grey PVC. The maze was placed in a quiet enclosure of the test room, and mice were habituated before the start. At the test start, mice were placed individually into the center platform facing an open arm and were allowed to move freely for 10 min [46]. The behavior was video recorded with a camera mounted above the maze. VideoMot2 (TSE Systems GmbH, Bad Homburg, Germany) was used for automatic tracking and analysis of arm entries, times in closed/open, and distances. The anxiety index was calculated as AI = (time in closed/total time in arms) − (time in open/total time in arms).

### Tail suspension test (TST)

In the TST, mice are suspended by the tail, which elicits defensive struggling and immobile hanging. The latter is often interpreted as resignation or depression resulting from an unsolvable and aversive situation [96], and the time of immobility is the primary readout. We used a self-constructed observation chamber which was divided into five compartments, separated by dark grey spacers, each compartment with a hook 30 cm above the floor. A widefield camera was mounted in front of a tripod to capture five mice simultaneously. The tail was fixed with adhesive tape to the hook, and each test lasted 10 min [1]. The video recordings were analyzed post hoc by using a computer key to measure the time and were done without knowledge of the group assignment.

### Sucrose preference and latency

Mice are highly addicted to sweet water. Compared with daily tap water volumes of about 4 ml, they may consume up to 16 ml sweet water (2% sucrose). High sucrose preference may be interpreted as consolation, or compulsiveness/addiction, depending on the extent and choices. To measure sucrose preference, a 2-choice test was used. Mice were housed in pairs per cage and habituated for 3 days by providing bottles of tap water on the right and left side of the cage. One bottle was then replaced with 5% sucrose in tap water, randomly on the right or left side. The volume intake was assessed by daily weighing the bottles. The latency to the first licking of sucrose water after an overnight water restriction was assessed in the home cage and in a new cage with new bedding. Mice were tested individually. The latency was assessed by observation with a stopwatch.

### Marble burying

The marble burying test has been used to assess stress or novelty-evoked anxiety (defensive burying or neophobia burying) [23, 61] or assessment of repetitive and compulsive behavior [98]. The test takes advantage of the normal spontaneous burying and digging behavior. Increased marble burying under stress reflects an inherent defensive response aimed at protecting from harmful objects. The behavioral meaning of marble burying is however debatable [23, 25]. For the MBT, 12 neutral 0.5-inch glass marbles were arrayed on the surface of clean, thick 7 cm bedding. The number of buried marbles (covered at least ¾ with bedding) was counted at 5 min, 10 min, 20 min, and at the end of a 30 min observation period. The MBT was done during and after completion of the CUMS protocol. The 30-min end point of “MBT-during-CUMS” was used for lipid association analyses. The investigator was unaware of the group assignment.

### Tissue and blood sampling

Mice were euthanized by CO2 in a chamber with adjustable stepwise increasing flow. Blood samples were collected in 500 µl K3-EDTA microtubes (Microvette Sarstedt) after cessation of respiration via cardiac puncture with a 27G needle attached to a 1-ml syringe. The samples were immediately centrifugated in a mini-tabletop centrifuge at 2000 g for 3 min; plasma was transferred into microtubes using a 100-µl pipette and directly frozen in liquid nitrogen and kept at − 80 °C until analysis.

The brain was rapidly removed; the olfactory bulb was removed (discarded); the cerebellum collected, weighed, and frozen in liquid nitrogen; the brain was cut sagittal; the halves unfolded, and then, regions were collected from rostral to dorsal: orbitofrontal and dorsal prefrontal cortex, striatum including nucleus accumbens, hippocampus, thalamus, hypothalamus, and midbrain. All samples were weighed on a precision scale and directly frozen in liquid nitrogen and kept at − 80 °C until analysis. Lipid concentrations are normalized on mg of tissue. Orbitofrontal and dorsal prefrontal cortex samples were analyzed separately, but concentrations were then averaged to get one PFC value per mouse to reduce brain sites and gain power.

### Analysis of lipid signaling molecules

Bioactive lipids including sphingoid bases and ceramides, lysophosphatidic acids (plasma only), and endocannabinoids (eCBs) were analyzed in plasma and in brain tissue homogenates using liquid–liquid-extraction (LLE) followed by liquid chromatography-electrospray ionization-tandem mass spectrometry (LC–ESI–MS/MS) as described in detail in a previous study [8]. Briefly, brain tissue samples were homogenized in ethanol:water (1:3, v/v) using a Mixer Mill MM400 (Retsch, Haan, Germany). Afterwards, brain tissue homogenates as well as plasma samples were extracted using an LLE protocol as described in detail in the previous publication. Sphingolipids were separated using an Agilent 1200 HPLC system equipped with a Zorbax C18 Eclipse Plus UHPLC column (50 × 2.1 mm, 1.8 µm, Agilent Technologies, Waldbronn, Germany). The analysis of LPAs (plasma only) was done on the same HPLC system using a Luna C18 column (50 × 2.0 mm, 5 µm, Phenomenex, Aschaffenburg, Germany). For the chromatographic separation of endocannabinoids, an Agilent 1290 Infinity I UHPLC system equipped with an Acquity UPLC BEH C18 UPLC column (100 × 2.1 mm, 1.7 µm, Waters, Eschborn, Germany) was used. The quantification of all analytes was performed using a hybrid triple quadrupole-ion trap mass spectrometer QTRAP 5500 or 6500 + (Sciex, Darmstadt, Germany) equipped with a Turbo-V-source operating in positive ESI mode for sphingolipids and endocannabinoids and in negative ESI mode for LPAs.

Quality control samples of three different concentration levels (low, middle, high) were run as initial and final samples of each run. For all analytes, the concentrations of the calibration standards, quality controls, and samples were evaluated by Analyst software 1.6 and MultiQuant software 3.0 (Sciex, Darmstadt, Germany) using the internal standard method (isotope-dilution mass spectrometry). Calibration curves were calculated by linear or quadratic regression with 1/x weighting or 1/ × 2 weighting.

To assess putative biases caused by sample sequence, concentrations were plotted versus analysis number. For ethanolamide endocannabinoids (AEA, OEA, PEA), linear regression analysis revealed a shallow but significant linear raise (significantly different from zero) with the sample number. The concentrations were therefore adjusted according to the analysis sequence number and the slope of the regression line. There was no effect of sample sequence for any of the other analytes.

### Statistics

Lipid concentrations are presented as scatter plots with mean ± standard deviation (SD) or box-scatter plots, where the box is the interquartile range and the whiskers show minimum to maximum, or the 95% confidence interval (CI), specified in the figure legend. Data were analyzed with SPSS 29, Origin Pro 2024, and GraphPad Prism 9.0. Principal component analysis (PCA) and partial least square analysis (PLS) were used to reduce dimensionality and identify the factors which contributed most to the difference between treatment groups and brain sites. In addition, linear canonical discriminant analysis (DA) was used to assess the predictability of group membership based on DA scores. DA was performed without and with bootstrapping, the latter using a stratified random sampling approach considering gender and age, and 100 iterations.

Lipid concentrations and behavioral readouts were compared between groups using analyses of variance (two-way ANOVA for brain site X treatment (CUMS or control), or *t*-tests according to the data subgroup structure and distribution. In case of significant results of ANOVAs, treatment groups were compared per brain site using *t*-tests comparing CUMS versus control. *P*-values were adjusted according to Šidák for multiple comparisons or were not adjusted if only two groups were compared. For behavioral time course data, 2-way ANOVA for repeated measurements “time” X “group” was used. The alpha level was set at 0.05 for all comparisons and asterisks in the figures refer to adjusted *P*-values.

For cluster analyses and polar plots, lipid concentrations were normalized to the median of all samples of the respective lipid to allow for a combined analysis and presentation. For correlation plots, lipids and behavioral readouts were scaled, and correlations are coded by color (blue negative correlation, red positive correlation) and bubble size according to the adjusted R-square. Further analyses consisted of multiple regression analyses to reveal associations between behavioral readouts and lipids that were different between CUMS and control mice at one or more brain sites. The regression analysis only included the CUMS groups based on the hypothesis that an increase or decrease of the respective lipid would be associated with a change of behavior rather than that the lipid concentration as such would predict a behavior. There was no association of behavior for any lipid in the control group.

## Results

### CUMS evoked mild weight loss and anxiety

**Fig. 1:**
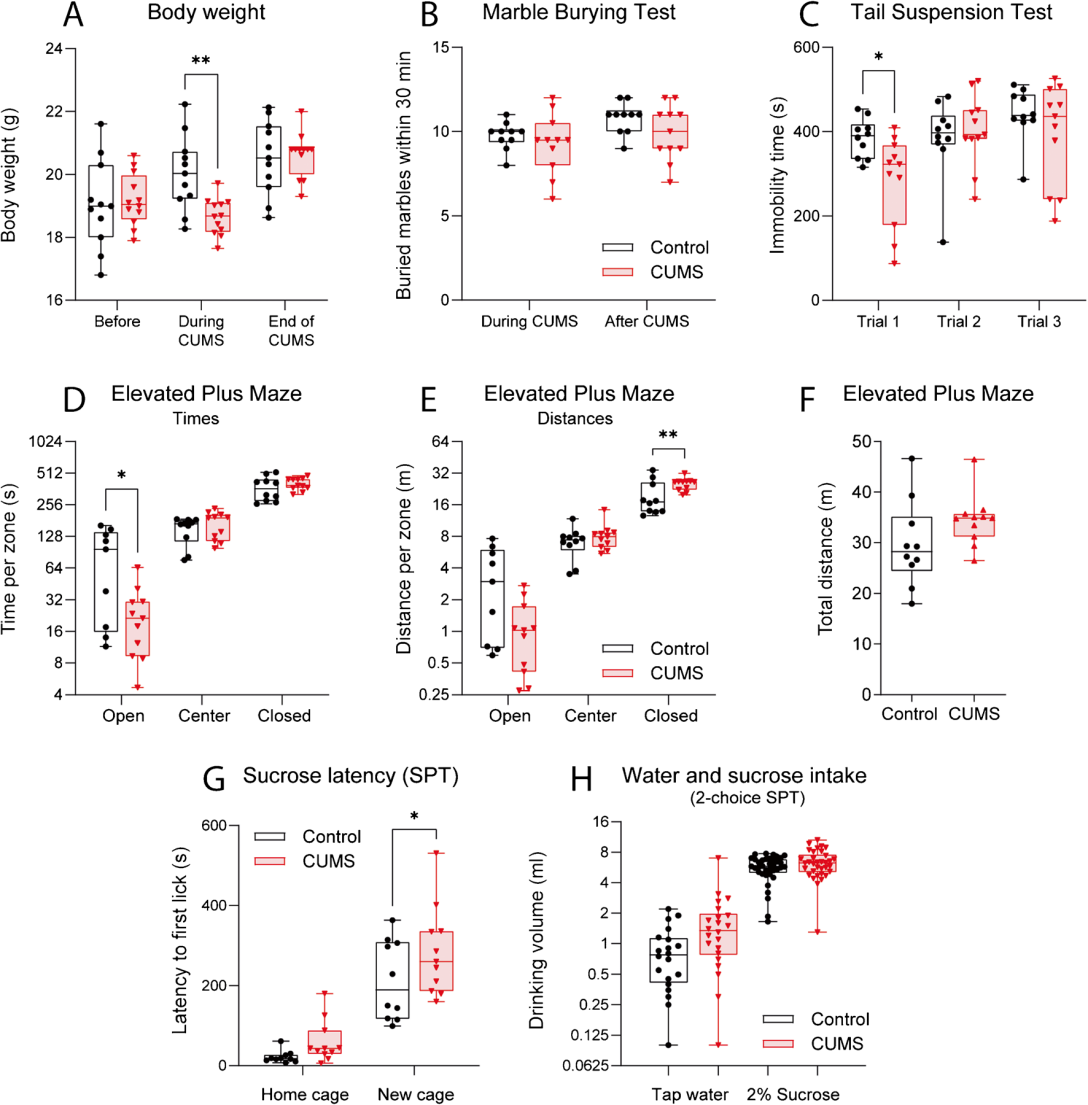
Behavior in a chronic unpredictable mild stress (CUMS) model of chronic environmental stress in mice. CUMS mice (*n* = 11) were exposed to mild daily stressors including cage switch, itch, cold exposure, predator odor, headache, no bedding, wet bedding, lights on overnight, no food overnight, no water overnight, restraint, cage shaking, tilted cage, or ultrasound noise for 6 weeks. Control mice (*n* = 10) were kept in neighboring cages without stress. Behavioral tests were done in the final CUMS week or at the end of the CUMS protocol. **A** Body weight before, during, and at the end of the CUMS protocol. **B** Numbers of buried marbles in the marble burying test (MBT, 12 marbles provided) within 30 min during and at the end of the CUMS protocol. **C** Immobility time in the tail suspension tests (TST) in three trials in the last 3 days of the CUMS protocol, one trial per day. **D**–**F** Path lengths (**D**) and times spent (**E**) in open and closed arms and the center compartment in elevated plus maze (EPM) test, and total distances traveled during the EPM observation of 10 min (**F**). **G** Latency to first licking from a standard bottle providing sweet water (2% sucrose in water) in the home cage and in an unfamiliar cage. **H** Water and sucrose-water intake (ml) in a 2-choice sucrose preference test (SPT) in home cages. The boxes show the interquartile range; the line is the median; the whiskers show minimum to maximum; scatters represent mice. Data were submitted to 2-way ANOVA (group X time, or group x EPM arm, etc.) and subsequent post hoc *t*-tests comparing CUMS versus control. Total EPM distances (**F**) were compared with a 2-sided unpaired Student’s *t*-test. The asterisks show significant differences between CUMS and control (*P < 0.05; **P < 0.01)

**Fig. 2:**
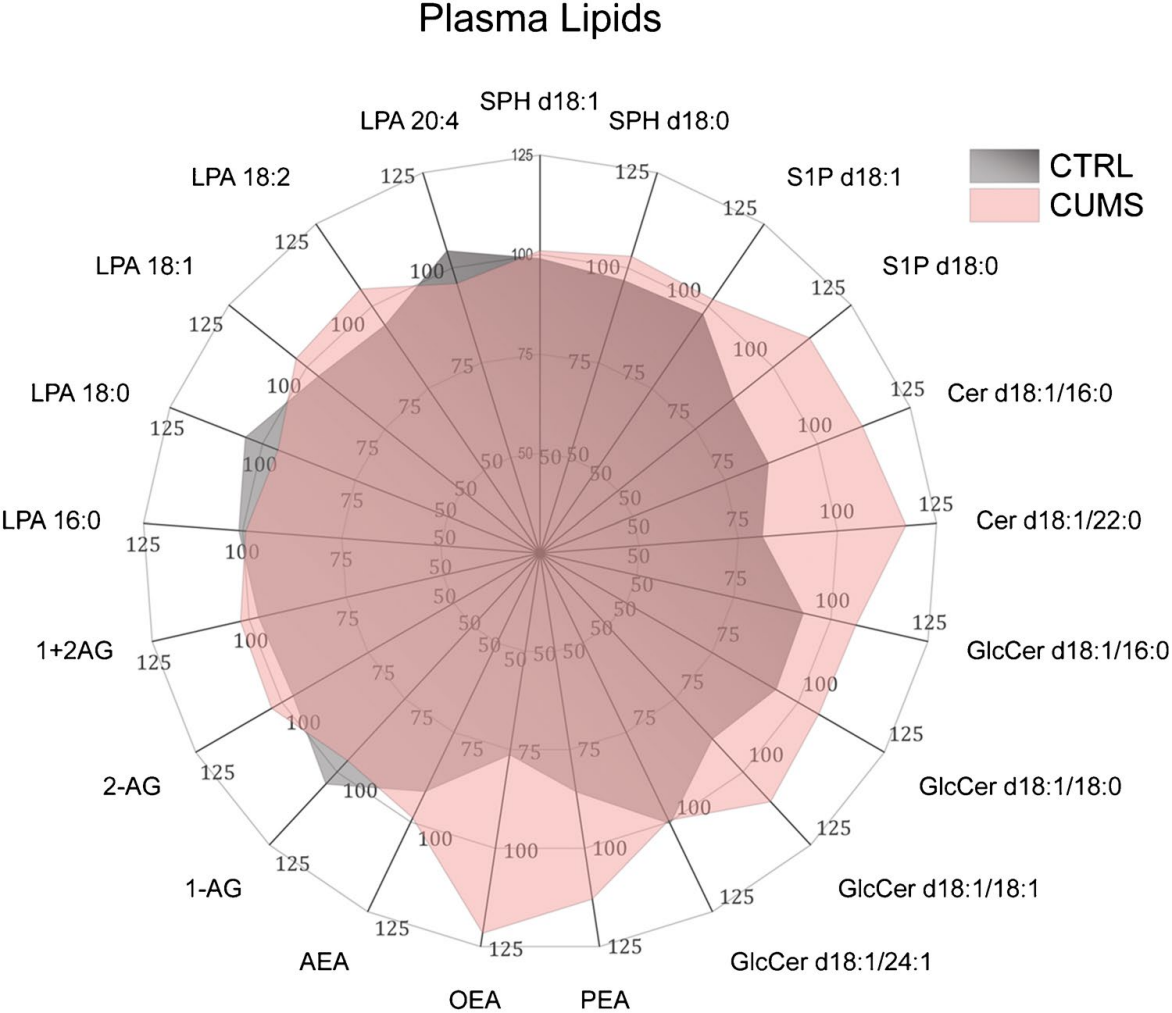
Circular map of CUMS-evoked changes of plasma lipids. Plasma concentrations were obtained by targeted UPLC-MS/MS, and data are revealed as percentages of the median concentration of the respective lipid, which was set to 100%. The graphs show the group averages. Abbreviations: AEA, anandamide; OEA, oleoylethanolamide; PEA, palmitoylethanolamide; AG, arachidonoylglycerol; Cer, ceramides; GlcCer, glucosylceramides; LPA, lysophosphatidic acid; SPH, sphingoid base (sphingosine d18:1, sphinganine d18:0); S1P, sphingosine/anine-1-phosphate

As expected, CUMS was associated with mild effects on health and behavior (Fig. 1A–H) manifesting in a mild reduction of body weight (Fig. 1A), an increase of plasma ceramides (Fig. 2), and a decrease in the time spent in open arms of the EPM (Fig. 1D, E), which may be interpreted as anxiety-like behavior. In addition, the latency to first licks of sweet water was increased in an unfamiliar novel cage as compared to controls (Fig. 1G), but sucrose intake was not different (Fig. 1H). The behavior agrees with anxiety-like behavior (“neophobia”) rather than “depression.” There was no difference in marble burying behavior (Fig. 1B) and total distance moved in maze tests (Fig. 1F). In the TST, immobility time was reduced in the first trial showing that CUMS mice spent more time struggling but was not consistently maintained upon repeated testing (Fig. 1C).

Plasma concentrations of endocannabinoids and sphingolipids were analyzed to assess the systemic metabolic impact of CUMS (Fig. 2). Ceramides were increased, particularly Cer d18:1/16:0 and Cer d18:1/22:0 which agrees with the expectation that stress disrupts metabolic homeostasis. High plasma concentrations of ceramides have been associated with obesity, diabetes [87], and mental disease (major depression, bipolar disorder) [8]. In addition, endocannabinoids, OEA, and PEA were increased, likely elicited by the loss of body weight during CUMS and favoring rapid body weight regain after CUMS (samples were taken after BW recovery) [36].

### Brain regions have site-specific lipid patterns

**Fig. 3:**
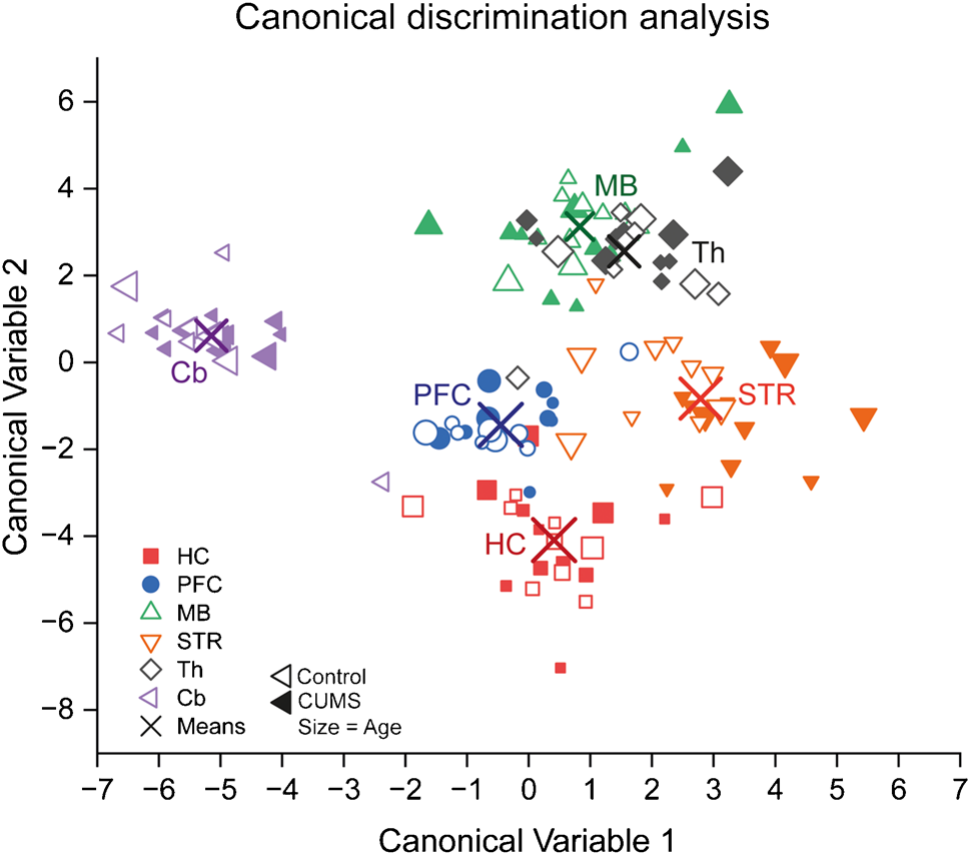
Canonical linear discrimination analysis of brain lipids in CUMS and control mice. The analysis was set to discriminate brain regions using concentrations of 14 lipid species (endocannabinoids, sphingoid bases, ceramides, and hexosylceramides) of CUMS mice (*n* = 11) which was used as the training set and applied to controls. The bubble plot shows the scores of the canonical discrimination variables 1 versus 2. Brain sites are color coded; closed symbols show CUMS mice; the bubble sizes represent mouse age. Abbreviations: Cb, cerebellum; HC, hippocampus; MB, midbrain; PFC, prefrontal cortex; Th, thalamus; STR, striatum

Lipid species of four classes (4 × endocannabinoids, 4 × sphingoid bases, 4 × ceramides, 5 × hexosylceramides) of seven brain sites were submitted to linear canonical discrimination analysis to reveal brain site-specific patterns and effects of CUMS (Fig. 3). Scatter plots of the discriminant scores (canonical variable 1 versus 2) show that the lipid patterns of the midbrain (MB) and thalamus (Th) are similar, that the cortical patterns of PFC and hippocampus are closely related, and the striatum is highly variable in between. The cerebellum is unique in its lipid patterns. The discrimination task was to separate sites, and CUMS levels were used for learning and applied to controls. The analysis shows that the patterns apply to both groups in agreement with the expectation of the model, which was meant to cause mild stress but no profound disruption.

**Fig. 4:**
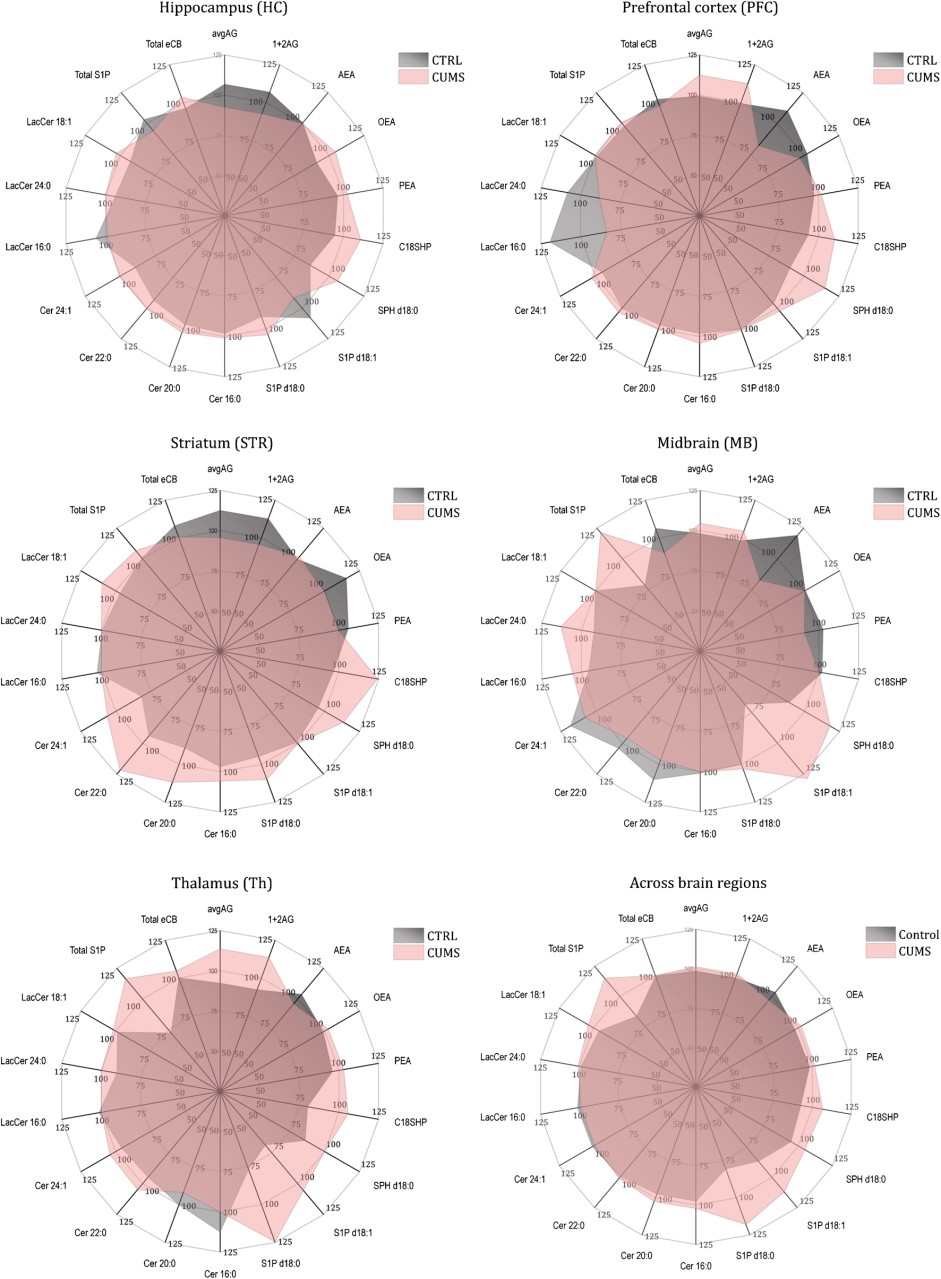
Circular map of CUMS-evoked changes of brain lipids at different brain regions. Tissue concentrations were obtained by targeted UPLC-MS/MS, and data are revealed as percentages of the median concentration of the respective lipid at the respective site, which was set to 100%. The graphs show the group averages. Abbreviations as in Fig. 2

The effects of CUMS however do reveal at the level of individual lipid species and are site-specific. In the first set of analyses presented as circular plots (Fig. 4), data were transformed to percentages versus the overall median to visualize the effect of CUMS. From the forebrain to the midbrain (top to bottom subpanels), the CUMS group impresses with reduced anandamide and lactosylceramides in PFC, an increase of sphingoid bases and ceramides in the striatum, and a further increase of sphingoid bases (but not ceramides) in the thalamus and midbrain. Across brain regions, S1P d18:1 (sphingosine-1-phosphate) and S1P d18:0 (sphinganine-1-phosphate) are higher in the CUMS group.

### Correlation of lipids across brain regions with behavioral readouts

**Fig. 5:**
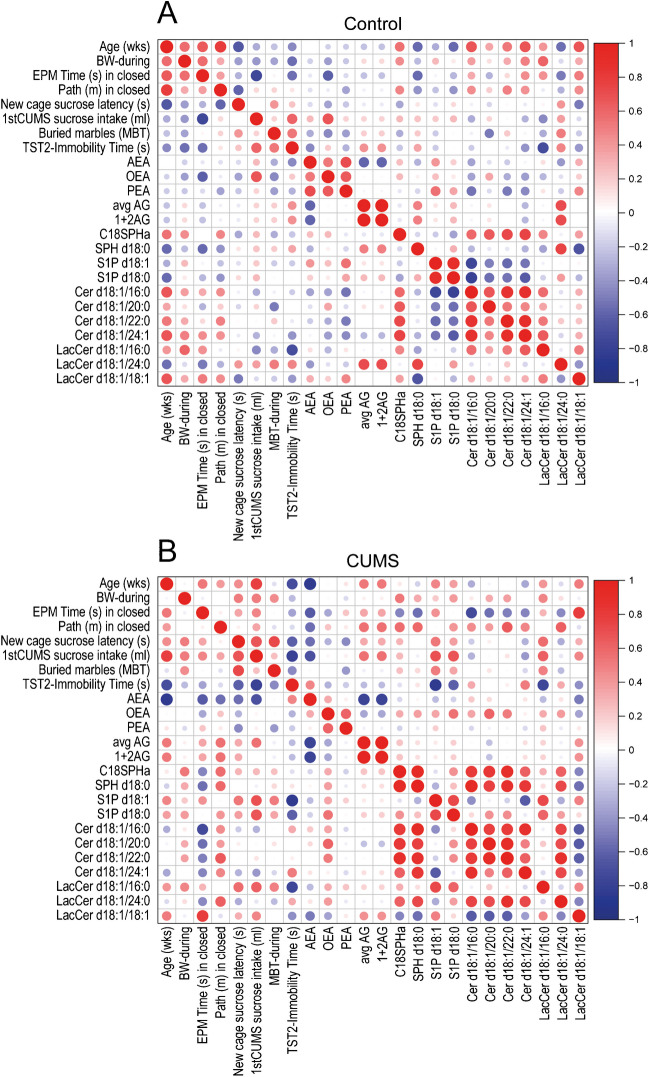
Correlation plots showing associations of brain lipids across sites and behavior. **A** Correlation plots of control mice. **B** Correlation plot of CUMS mice. The correlation is color coded as revealed in the scale bar. Red denotes high positive correlation and blue high negative correlation. In addition, the size of the dots shows a correlation (positive or negative). Please note the strong differences of the S1P d18:1 and S1P d18:0 correlations and of LacCer (lactosylceramides)

Lipid levels across brain regions (i.e., the average of all sites for each mouse), body weights, and behavioral readouts were submitted to correlation analyses and plotted as correlation plots (Fig. 5) to reveal if and how brain lipids or behavioral features were associated with each other. The analysis was done for control and CUMS separately to compare patterns. The CUMS correlation map has more positive (red) and negative (blue) correlations. Again, S1P and LacCer show the strongest differences. In controls, S1P is negatively correlated with ceramides, which is lost or inverted in CUMS. In CUMS, there are strong negative correlations of AEA with behavior which is not evident in controls.

**Fig. 6:**
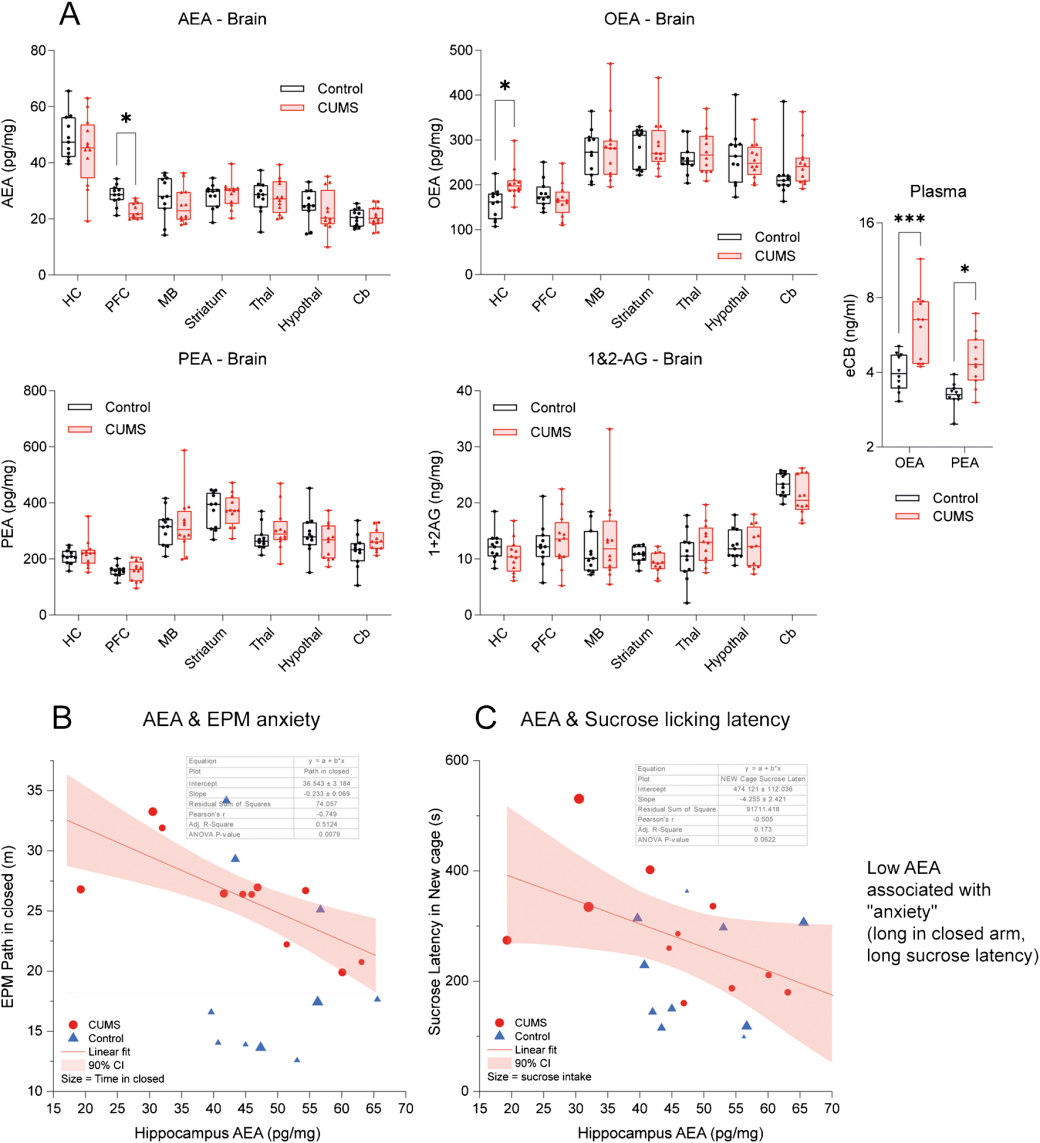
Endocannabinoids at different brain sites and association of AEA with behavior of anxiety. **A** Box/scatter plots show endocannabinoid concentrations in different brain regions and OEA and PEA in plasma. The boxes show the interquartile range; the line is the median; whiskers show minimum to maximum; scatters represent mice. Data were submitted to 2-way ANOVA (group X site) and subsequent post hoc *t*-tests comparing CUMS versus control. The asterisks show significant differences between CUMS and control (*P < 0.05). **B**, **C** Linear regression analysis of AEA concentrations in the hippocampus (HC) with behavioral readouts of anxiety in the EPM (path in the closed arm) and in the sucrose latency test in an unfamiliar cage. Table inserts show the regression parameters and *P*-values. The bubble size represents the time in closed arms (EPM, **B**) or the sucrose-water consumption (drinking volumes) in **C**. The line shows the linear fit; the bands show the 90% confidence intervals. Abbreviations: AEA, anandamide; OEA, oleoylethanolamide; PEA, palmitoylethanolamide; AG, arachidonoylglycerol; Cb, cerebellum; HC, hippocampus; MB, midbrain; PFC, prefrontal cortex; Th, thalamus; STR, striatum

**Fig. 7:**
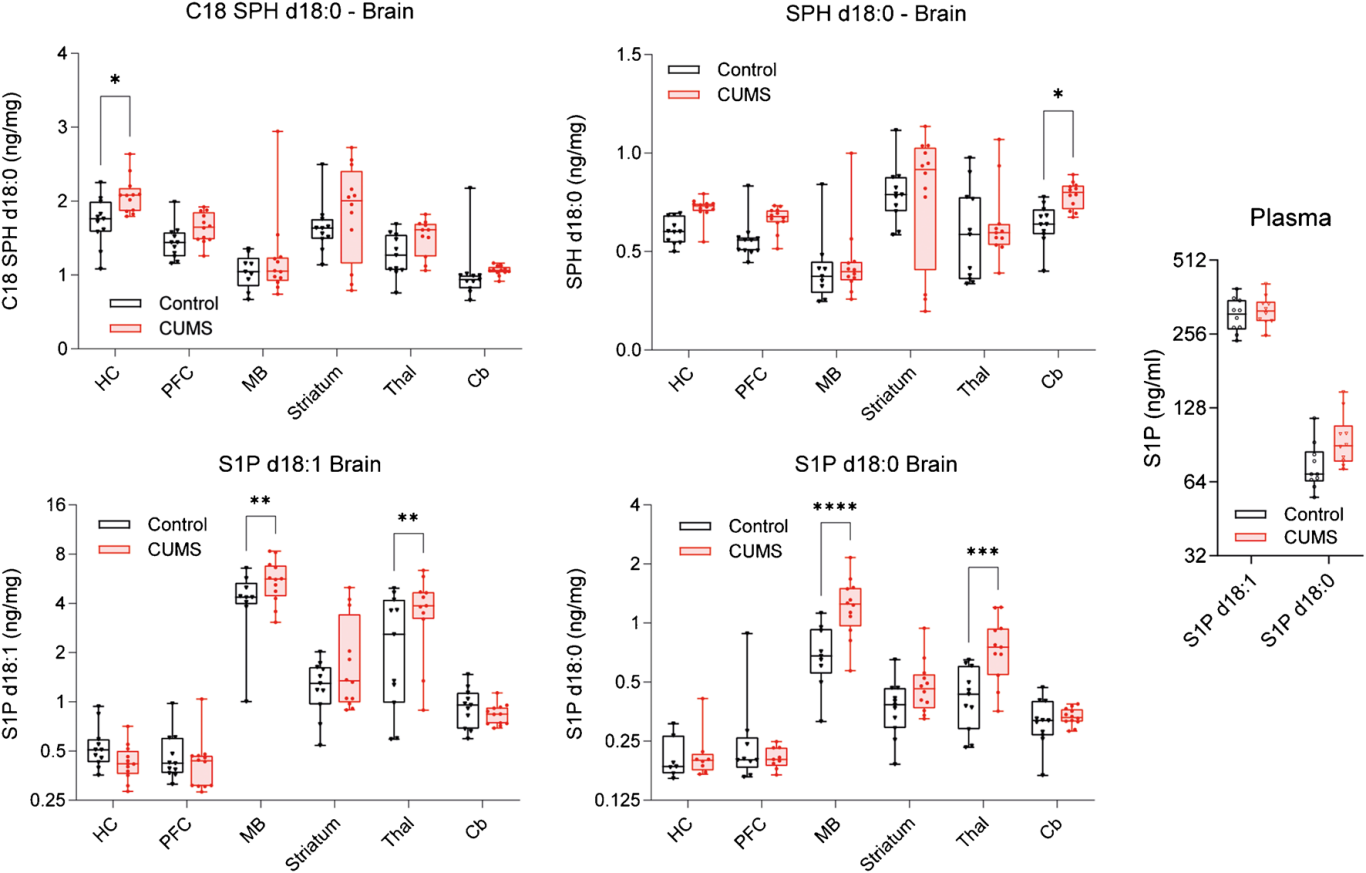
Sphingosine, sphinganine, and S1P at different brain sites. Box/scatter plots show concentrations of sphingoid bases (sphingosine and sphinganine) and phosphorylated sphingoid bases (S1P d18:1, S1P d18:0) in different brain regions and S1P in plasma. The boxes show the interquartile range; the line is the median; whiskers show minimum to maximum; scatters represent mice. Data were submitted to 2-way ANOVA (group X site) and subsequent post hoc *t*-tests comparing CUMS versus control. The asterisks show significant differences between CUMS and control (*P < 0.05, ** < 0.01, *** < 0.001, **** < 0.0001). Brain site abbreviations as in Fig. 6

**Fig. 8:**
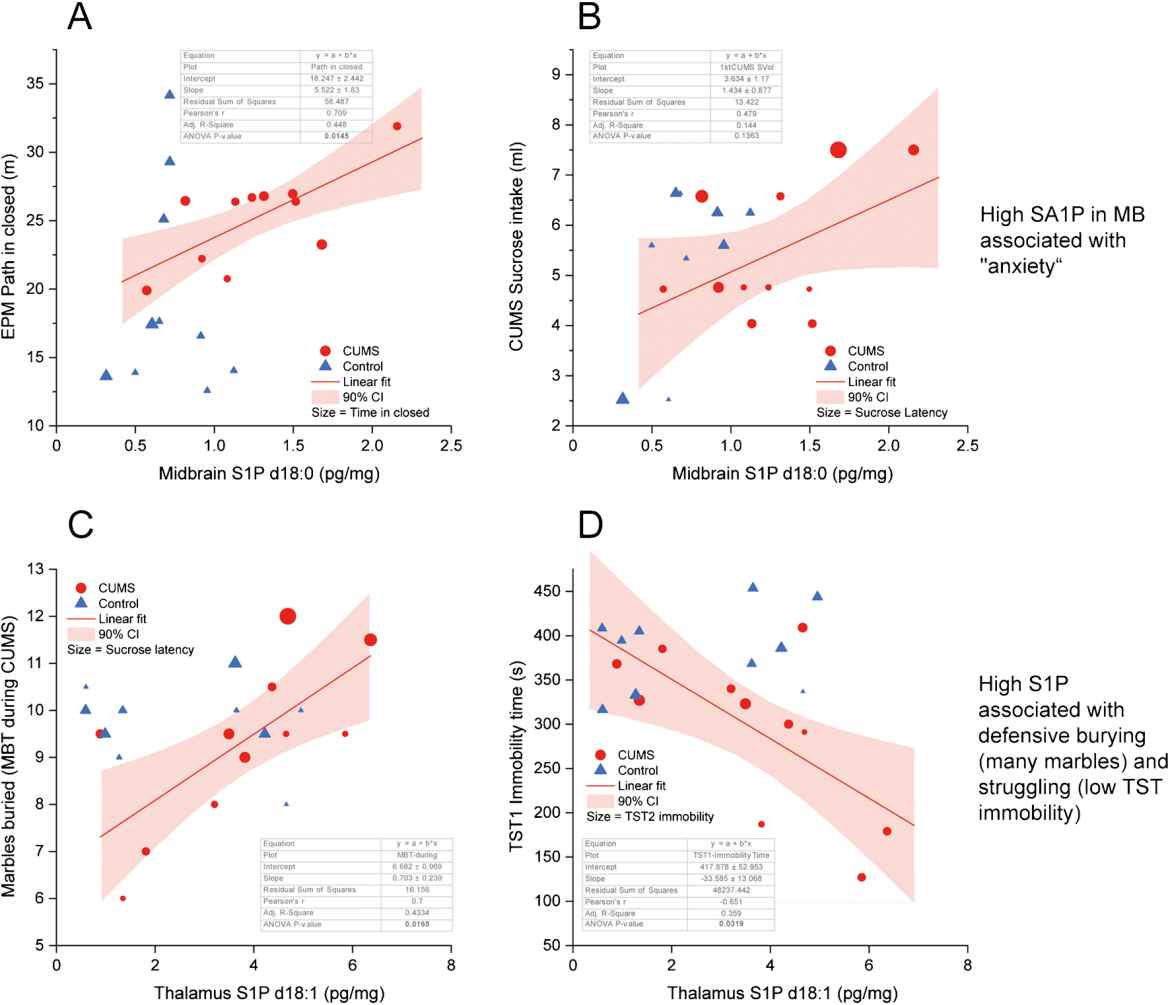
Associations of S1P in the thalamus with behavioral readouts of stress resilience. **A**, **B** Linear regression analysis of S1P d18:0 (sphinganine-1-phosphate) concentrations in the midbrain with EPM paths in the closed arm (but not with time in the closed arm, bubble size) and sweet water consumption (n.s.). There was no association with sucrose-water latency (bubble size). **C**, **D** Linear regression analysis of S1P d18:1 (sphingosine-1-phosphate) concentrations in the thalamus with the number of buried marbles in the MBT and the immobility time in the tail suspension test (TST). The higher the S1P, the more marbles were buried, showing strong defensive efforts. In agreement, the higher the S1P levels, the lower the immobility time, which is the inverse of the struggling time. Strong struggling indicates high defensive efforts. Table inserts show the regression parameters and *P*-values. The line shows the linear fit; the bands show the 90% confidence intervals

**Fig. 9:**
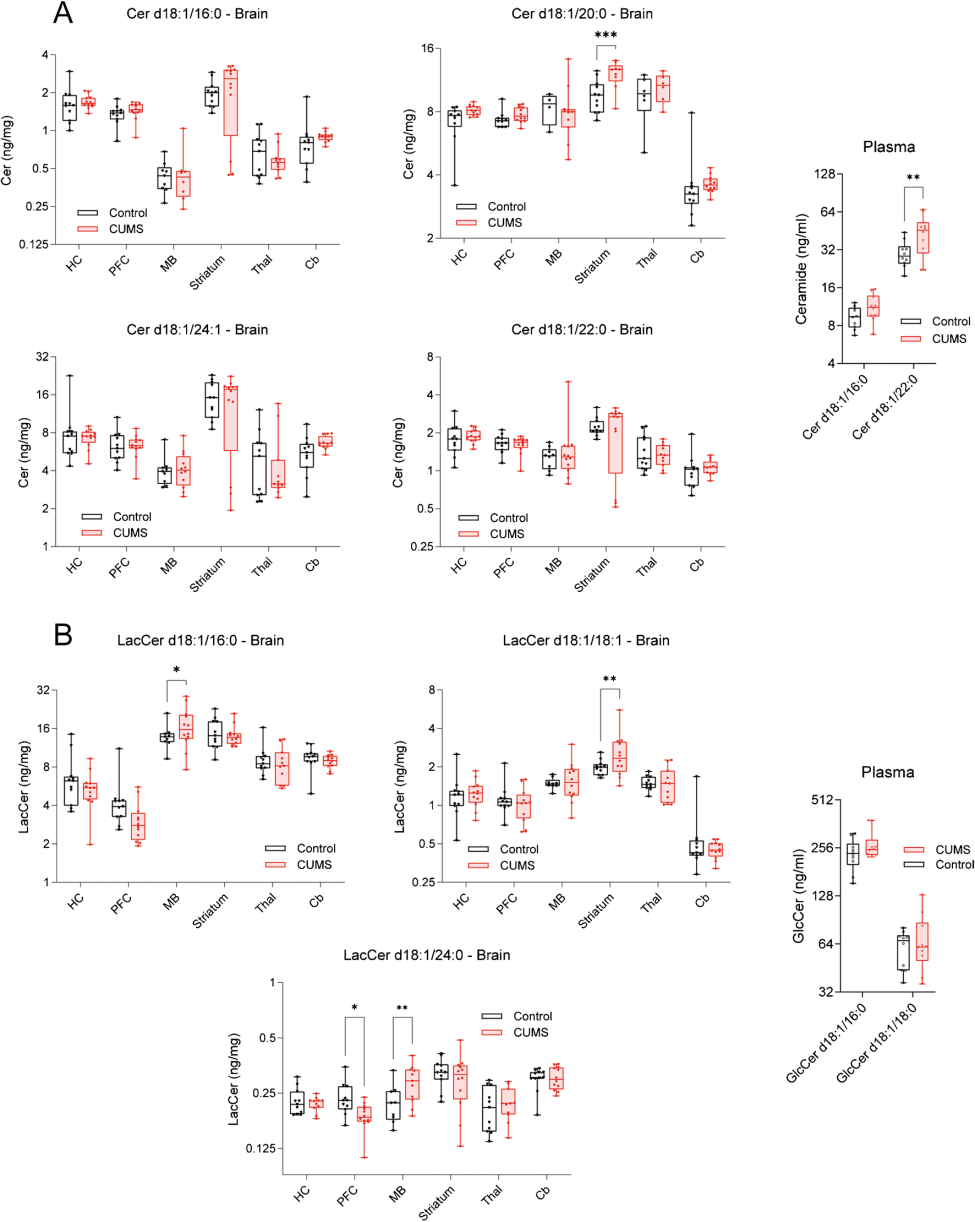
Ceramides and hexosylceramides at different brain sites and in plasma. **A**, **B** Box/scatter plots show concentrations of ceramides (**A**) and hexosylceramides (**B**) in different brain regions and in plasma. The boxes show the interquartile range; the line is the median; whiskers show minimum to maximum; scatters represent mice. Data were submitted to 2-way ANOVA (group X site) and subsequent post hoc *t*-tests comparing CUMS versus control. The asterisks show significant differences between CUMS and control (*P < 0.05, ** < 0.01, *** < 0.001). Brain site abbreviations as in Fig. 6. Lipids: Cer, Ceramides; LacCer, lactosylceramides; GlcCer, glucosylceramides

Correlation maps, circular lipid plots, and behavior suggested two hypotheses: (i) anandamide (AEA) deficiency in the hippocampus and cortex evoked by CUMS is associated with anxiety, and (ii) S1P in subcortical structures is associated with defensive and combative depression-averting behavior. To address the hypothesis, individual site-specific lipid concentrations were analyzed in detail in associations with behavioral features using multiple regression analyses (Figs. 6, 7, 8, and 9).

### CUMS-evoked low AEA in the cortex associated with anxiety

AEA was significantly reduced in the PFC in CUMS mice versus controls, and OEA was increased in the hippocampus (Fig. 6A). As revealed in Fig. 2, OEA and PEA were increased in the plasma of CUMS versus control mice. AEA is a canonical cannabinoid receptor ligand, and therefore, associations with behavior were primarily assessed for AEA. The hippocampus was chosen as the primary site (although n.s.) because the HC concentrations in CUMS mice showed a broad range suggesting that HC-AEA may reflect the individual susceptibility to CUMS-evoked behavior. Linear regression analyses revealed that low AEA in hippocampus (or PFC not shown) was significantly associated with long distance (Fig. 6B) (or long time, not shown) in the closed arms in EPM. The lower AEA, the more time was spent in the closed arm, suggesting that low AEA increased anxiety or reduced curiosity. Low AEA in the hippocampus was also associated with a long sucrose latency in an unfamiliar cage, which however did not reach statistical significance (*P* 0.062).

### CUMS-evoked high S1P in the thalamus and midbrain associated with TST struggling and MBT

S1P d18:1 (sphingosine-1-phosphate) and S1P d18:0 (sphinganine-1-phosphate) were increased in the midbrain and thalamus in CUMS mice (Fig. 7). In addition, non-phosphorylated sphinganine was increased in the hippocampus, PFC, and cerebellum in CUMS versus control. Linear regression analyses (Fig. 8) show that high S1P d18:0 (sphinganine-1-phosphate) is associated with long paths in the closed EPM arms (Fig. 8A). However, there was no association with time spent in closed arms (not shown, linear adj R-square − 0.111; ANOVA *P*-value 0.958) showing that mice with high S1P d18:0 in midbrain were more active within the closed arms. There was no association of S1P d18:0 with sucrose latency (Fig. 8B). S1P d18:1 in the thalamus (or across regions, not shown) was associated with a high number of buried marbles in the MBT (Fig. 8C) and high struggling time in TST (which is the inverse of the immobility time) (Fig. 8D).

Although previous studies in mice suggest that ceramides are particularly important for stress-evoked depression-like behavior and plasma ceramides agree with the expected stress-evoked increase, there were no consistent between group differences and no significant association between plasma and across-brain ceramides levels. Notably, ceramides were high in the striatum in both groups compared with other brain regions.

## Discussion

The present study shows that CUMS in mice leads to mostly subtle site-specific adjustments of endocannabinoids, particularly a loss of AEA in the PFC and an increase of sphingolipids, particularly S1P in the midbrain and thalamus. Low AEA in PFC and hippocampus are associated with anxiety-like behavior, i.e., preference of dark zone in EPM and long sucrose latency in an unfamiliar cage. These results agree well with previous studies of chronic stress in mice where AEA was found reduced in the brain, albeit not specifically in cortical structures but throughout studied brain regions [5, 7, 49, 57]. Other studies using electrophysiology and CB1 receptor knockout have suggested that specifically the endocannabinoid systems of the hippocampus and amygdala have a key role in anxiety extinction [11, 50, 88] and stress relief [67]. Human imaging studies using cannabinoid radioligands reveal increases in free CB1 binding capacity in stress-associated diseases such as posttraumatic stress disorder suggesting a relative deficiency of endocannabinoids at affected sites [73, 79]. Brain samples in our study were collected directly at the end of the CUMS protocol, hence not allowing mice to learn that the stress period was finished. Therefore, we assume that the brain was captured in a state of heightened alertness, unease, and fear for the next stressor and not in a state of stress relief, which is expected to switch the brain into reward mode [32, 33] associated with an increase of endocannabinoids and enhancement of dopamine release from crucial reward sites of the mesolimbic system [68, 71, 80, 105]. The repeated experience of stress relief was shown to increase stress resilience and prevent depressive symptoms in mice [32].

Resilience in mice is mostly inferred from non-occurrence of stress-evoked fear, i.e., as a negative readout, and the meaning of specific behaviors is context-sensitive and complex. In the present study, we interpreted the struggling behavior in the TST and the marble burying behavior in the MBT as positive readouts of active defensive behavior and hence resilience [24, 25], as opposing to depression that would manifest as immobility, low locomotion, reduced feeding, or reduced nest building [85, 96]. This interpretation is supported by studies which use the immobility time in TST or small numbers of buried marbles as indicators of depression [22, 38, 39, 110]. The MBT is particularly controversial. Digging and burying of noxious, harmless, or rewarding objects is part of normal food searching and nest building behavior, and it is expressed in home cages without stress and under anxiogenic circumstances for example imposed by exposure to noxious objects or predator odor [25]. Defensive burying has been defined as the process of moving bedding material to cover harmful stimuli such as sources of electrical foot shock, and it is used as a measure of aversive anxiety [15, 61, 75]. In addition, burying of novel objects under stressful conditions is considered neophobia but mostly does not well agree with other readouts of novelty-evoked fear [25], and excessive marble burying was suggested to reveal nonfunctional repetitive behavior analogous to behavioral symptoms of obsessive–compulsive disorder [23, 30, 109], impulsivity, autism, and dementia, however with low predictive value for therapeutics of such disorders [40]. Hence, as a stand-alone test, MBT offers alternative interpretations. In the present study, the numbers of buried marbles in CUMS mice were more variable but not significantly different from those of control mice but were positively correlated with the struggling time in the TST, which is the inverse of the immobility time, and with S1P d18:1 and S1P d18:0 levels in the thalamus and across brain regions. It is not much known about the putative functions of S1P in the brain for adjustments of the brain towards chronic stress and coping with chronic stress. However, one study revealed that fingolimod increased stress resilience (i.e., prevented stress-evoked depression-like behavior) in a model of chronic unpredictable stress via activation of S1PR3 in the medial prefrontal cortex [19, 21]. Once phosphorylated, fingolimod is an S1P receptor agonist [45]. However, it is mostly recognized for opposite net effects, i.e., reduction of S1P signaling resulting from receptor downregulation. The latter effect prevents T-cells from egress of secondary lymphoid organs, hence explaining its therapeutic efficacy in autoimmune-mediated diseases, particularly preventing relapse in multiple sclerosis [17]. Major depression, schizophrenia, PTSD, and other psychiatric diseases are believed to be contributed or sustained by inadequate immune activation [26, 55, 77, 92]. Hence, many studies assessed the efficacy of fingolimod in experimental models of such psychiatric diseases mostly with some therapeutic benefit for fingolimod-treated mice [27, 28, 45, 65, 112], which was however not confirmed in clinical studies [60]. Considering differences in treatment schedules in mice and psychiatric patients, it may be suggested that long-term fingolimod leads to receptor downregulation in the brain similar to its regulation in immune cells and hence interferes with resilience-strengthening effects of endogenous brain S1P under stress conditions. Indeed, depression is frequent in MS patients receiving fingolimod, but switching to fingolimod from other disease-modifying drugs was reported to reduce depression at least temporarily [53]. Although chronic stress-evoked mental health issues are believed to be contributed by immune activation, we did not observe an increase of plasma S1P in CUMS mice. Instead, plasma lipids revealed an increase of ceramides, particularly Cer d18:1/22:0 which are increased in patients with major depression and bipolar disorder, again particularly Cer d18:1/22:0 [8, 9]. Ceramides were not associated with body weight presumably because time points of blood sampling and weight loss during CUMS did not match. Blood was obtained at the end of the CUMS protocol when the body weight was already restored. Nevertheless, alterations of plasma ceramides well agree with the concept of stress-evoked unfavorable metabolic effects which however did not correlate with behavioral readouts of “mental” health in our mice.

In summary, we show that CUMS in mice resulted in low AEA levels in cortical brain regions in correlation with anxiety-like behavior and high S1P levels in the midbrain and thalamus in correlation with defensive behavior. The results agree with previous studies of brain anandamide under stress and strengthen the idea that cannabinoids might be useful in certain cases of PTSD [18, 90]. S1P results are novel. They agree with the strengthening of resilience with short-term fingolimod treatment in mice but suggest that sphingosine kinase inhibitors that are under investigation for cancer and fibrosis [12, 44, 91, 111] might affect mental health, which has so far not been observed.

## Supplementary Information

Below is the link to the electronic supplementary material.

Supplementary file1 (PDF 379 KB)

## Data Availability

Data are included in the manuscript or supplement. Additional raw data are available upon reasonable scientific request from the corresponding author.

## Abbreviations

AG: Arachidonoylglycerol
AEA: Anandamide
Cer: Ceramides
LacCer: Lactosylceramides
GlcCer: Glucosylceramides
eCB: Endocannabinoid
SPH: Sphingolipids
S1P (S1P d18:1): Sphingosine-1-phosphate
SA1P (S1P d18:0): Sphinganine-1-phosphate
CUMS: Chronic unpredictable mild stress
EPM: Elevated plus maze
SPT: Sucrose preference tests
TST: Tail suspension test
ScrLat: Sucrose licking latency
OEA: Oleoylethanolamide
PEA: Palmitoylethanolamide
AG12: 1/2-Arachidonoylglycerol
SPH d18:0: Sphinganine
SPH d18:1: Sphingosine
SA1P (S1P d18:0): Sphinganine-1-phosphate
Cer (d18:1/16:0): Ceramide with 16 C
Cer (d18:1/18:0): Ceramide with 18 C
Cer (d18:1/20:0): Ceramide with 20 C
Cer (d18:1/22:0): Ceramide with 22 C
Cer (d18:1/24:0): Ceramide with 24 C
Cer (d18:1/24:1): Ceramide with 24 C, one unsat.
GlcCer (d18:1/16:0): Glucosylceramide, 16C
GlcCer (d18:1/18:0): Glucosylceramide, 18C
GlcCer (d18:1/24:1): Glucosylceramide, 24C, one unsat.
LacCer (d18:1/16:0): Lactosylceramide, 16C
LacCer (d18:1/18:0): Lactosylceramide, 18C
LacCer (d18:1/24:0): Lactosylceramide, 24C
LacCer (d18:1/24:1): Lactosylceramide, 24C, one unsat.
LacCer (d18:1/18:1): Lactosylceramide, 18C, one unsat.
LPA16:0: Lysophosphatidic acid 16C
LPA18:0: Lysophosphatidic acid 18C
LPA18:1: Lysophosphatidic acid 18C, one unsat.
LPA18:2: Lysophosphatidic acid 18C, two unsat.
LPA20:4: Lysophosphatidic acid 20C, four unsat.
BW: Body weight
LLOQ: Lower limit of quantification
ULOQ: Upper limit of quantification
QC: Quality control
IS: Internal standard
UHPLC: Ultrahigh performance liquid chromatography
m/z: Mass transition
ANOVA: Analysis of variance
ASCA: ANOVA-simultaneous component analysis
CI: Confidence interval
DA: Discriminant analysis
PLS: Partial least square analysis
PCA: Principal component analysis
MBT: Marble burying test
