# Supplementary material for: Brain region specific regulation of anandamide (down) and sphingosine-1-phosphate (up) in association with anxiety (AEA) and resilience (S1P) in a mouse model of chronic unpredictable mild stress
Source: Pflugers Arch. 2024 Aug 23;476(12):1863–80. doi: 10.1007/s00424-024-03012-0 (PMC11582197; doi:10.1007/s00424-024-03012-0)
Supplement: Supplementary file 1 — Supplementary file1 (PDF 379 KB) [file 424_2024_3012_MOESM1_ESM.pdf]

Experiment with CUMS and control group
Experiment only with CUMS group

Time	Monday	Tuesday	Wednesday	Thurday	Friday	Saturday	Sunday
08:00	Mice weighing	weigh bottles + switch side	weigh bottles + switch side	weigh bottles + switch side	New cage	New cage	
09:00					Mice hair collection		
10:00						Cage shaking (1h)	
11:00							
12:00							
13:00							
14:00							
15:00							
16:00							
17:00	2 % Sucrose vs water bottles				TMT odor overnight	No water overnight	Lights on overnight

Time	Monday	Tuesday	Wednesday	Thurday	Friday	Saturday	Sunday
08:00	Cage switching				New cage		
09:00		Tilted cage (3h)	No bedding 3h	Restraint 1 h			
10:00						Shaking 1h	
11:00							
12:00	Itch						
13:00							
14:00							
15:00	Cold exposure 20 min	No food overnight		Wet bedding overnight			
16:00							
17:00					Lights on overnight	No water overnight	Cage Switching

Time	Monday	Tuesday	Wednesday	Thurday	Friday	Saturday	Sunday
08:00	New cage			New cage			
09:00		Tilted cage 3h	Cold exposure 20 min				
10:00				Itch Cp 48-80			
11:00							
12:00							
13:00		Headache			Low dose LPS (100 µg/kg)	Itch	Itch
14:00							
15:00							
16:00							
17:00	Unreachable food overnight		Wet bedding overnight	No water over night	No food over night		No water overnight

Time	Monday	Tuesday	Wednesday	Thurday	Friday	Saturday	Sunday
08:00							
09:00	Headache		No bedding (3h)	4 x 30 min ultrasound	Tilted cage 3h	Cage shaking 1h	Restraint 1h
10:00		Cage Switching					
11:00							
12:00							
13:00							
14:00							
15:00	Cold exposure 20 min		Cage shaking 1h	Restraint 1 h	Itch Cp 48/80		
16:00							
17:00		Wet bedding overnight	No water overnight			No water overnight	No food overnight

Time	Monday	Tuesday	Wednesday	Thurday	Friday	Saturday	Sunday
08:00	Cold exposure 20 min	weigh bottles + switch side	weigh bottles + switch side	weigh bottles + switch side			
09:00							
10:00		No bedding 2h	Headache	Cage shaking 1h			
11:00							
12:00				Marble burying test	Marble burying test		
13:00		Itch Cp 48/80	Marble burying test				
14:00		Ultrasound 2h					
15:00							
16:00				1 h Restraint			
17:00	Wet bedding overnight	Lights on overnight	TMT odor (10 µl filter paper)				
	2 % Sucrose vs water bottles						

Time	Monday	Tuesday	Wednesday	Thurday	Friday	Saturday	Sunday
08:00		weigh bottles + switch side	weigh bottles + switch side	weigh bottles + switch side	New Cage	Cold exposure 20 min	
09:00							
10:00			Marble burying test	TST - Control group	TST - Control group	TST - Control group	
11:00							
12:00							
13:00				Sucrose latency - Unfamiliar CUMS Group	2 h Restraint		Headache
14:00			Sucrose latency - home cage			1 h no bedding	
15:00							
16:00				Cold exposure 20 min	Itch Cp 48/80		
17:00							
				Wet bedding overnight	No water overnight	Unreachable food overnight	Wet bedding overnight
	2 % Sucrose vs water bottles						

Time	Monday	Tuesday	Wednesday	Thurday	Friday	Saturday	Sunday
08:00		weigh bottles + switch side	weigh bottles + switch side	TST CUMS Group			
09:00							
10:00		TST CUMS Group	Marble burying test	2 h restraint			
11:00							
12:00	2 h Restraint			Mice weighing + hair collection			
13:00		Cold exposure 20 min	TST CUMS Group				
14:00			EPM CUMS Group	Tissue collection CUMS group			
15:00							
16:00			Sucrose latency-unfamiliar				
17:00	Unreachable food overnight	No bedding overnight	Wet bedding overnight				
	2 % Sucrose vs water bottles						

Time	Monday	Tuesday	Wednesday	Thurday	Friday	Saturday	Sunday
08:00	EPM Control Group						
09:00				Sucrose latency-home cage			
10:00	2 % Sucrose vs water bottles	weigh bottles + switch side	Marble burying test				
11:00				Mice weighing + Hair collection			
12:00							
13:00			Sucrose latency-unfamiliar	Tissue collection control group			
14:00							
15:00							
16:00							
17:00							
